# ESCRT Function in Cytokinesis: Location, Dynamics and Regulation by Mitotic Kinases

**DOI:** 10.3390/ijms151221723

**Published:** 2014-11-25

**Authors:** Musab S. Bhutta, Christopher J. McInerny, Gwyn W. Gould

**Affiliations:** Henry Wellcome Laboratory of Cell Biology, Davidson Building, Institute of Molecular, Cell and Systems Biology, College of Medical, Veterinary and Life Sciences, University of Glasgow, Glasgow G12 8QQ, UK; E-Mail: Musabsaeed@ymail.com

**Keywords:** endosomal sorting complex required for transport (ESCRT) protein, cytokinesis, Aurora, polo kinase, abscission

## Abstract

Mammalian cytokinesis proceeds by constriction of an actomyosin ring and furrow ingression, resulting in the formation of the midbody bridge connecting two daughter cells. At the centre of the midbody resides the Flemming body, a dense proteinaceous ring surrounding the interlocking ends of anti-parallel microtubule arrays. Abscission, the terminal step of cytokinesis, occurs near the Flemming body. A series of broad processes govern abscission: the initiation and stabilisation of the abscission zone, followed by microtubule severing and membrane scission—The latter mediated by the endosomal sorting complex required for transport (ESCRT) proteins. A key goal of cell and developmental biologists is to develop a clear understanding of the mechanisms that underpin abscission, and how the spatiotemporal coordination of these events with previous stages in cell division is accomplished. This article will focus on the function and dynamics of the ESCRT proteins in abscission and will review recent work, which has begun to explore how these complex protein assemblies are regulated by the cell cycle machinery.

## 1. Resolving the Bridge: Cleavage of the Intercellular Bridge Is a Multi-Step, Tightly Regulated Process

**Figure 1:**
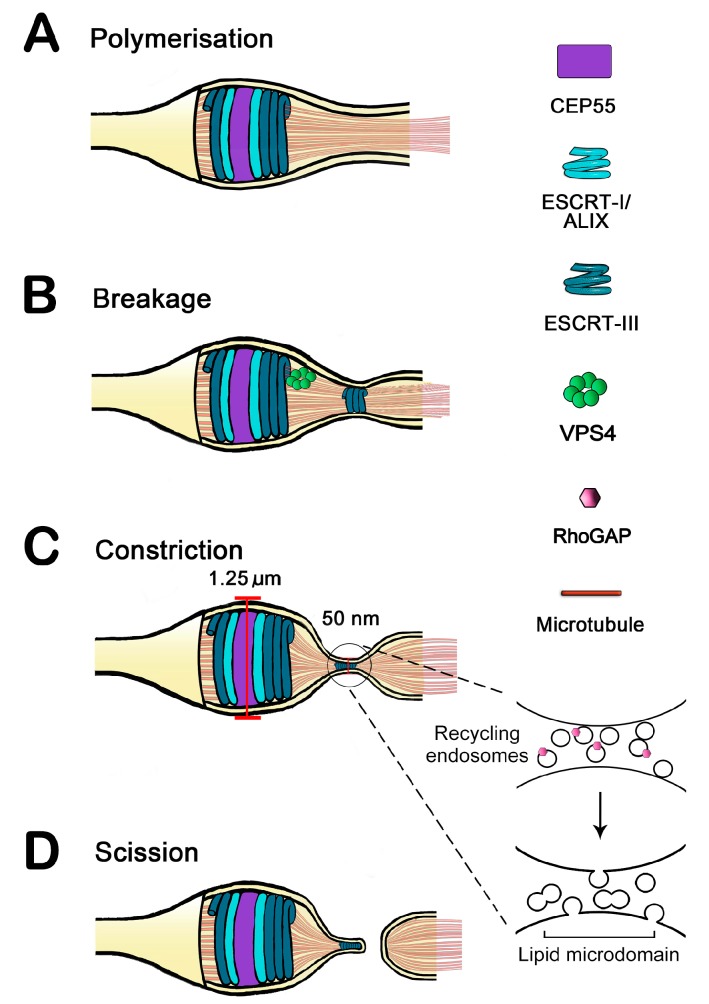
Schematic model for abscission in mammalian cells. In panel (**A**), CEP55 (Centrosomal protein of 55 kDa) has first recruited ESCRT-I components (Tumour susceptibility gene 101 (TSG101), apoptosis-linked gene 2-interacting protein (ALIX)) to the Flemming body, followed by the recruitment of ESCRT-III; (**B**) The Recruitment of the ATPase VPS4 to this assembly is proposed to mediate breakage or remodeling of the ESCRT-III, facilitating the appearance of ESCRT-III at the abscission zone (for details of different models for this event, see text); (**C**) Constriction at the secondary ingression site may be driven either by fusion of endosomal vesicles with the plasma membrane (inset), or by ESCRT-III interactions with the plasma membrane (see text). The possible role of lipid domains and the Rho GTPase activating protein (RhoGAP) (insert) is discussed in the text; and (**D**) Scission is thought to be driven by the ESCRT-III/VPS4 complex.

After furrowing is complete, the newly formed daughter cells remain attached by a thin intercellular bridge; Abscission completes the formation of the daughter cells by cleaving this bridge (Figure 1) [1,2]. This requires the assembly of the abscission machine with absolutely precise spatial and temporal coordinates. The final step, cleavage of the membrane bridge, is mediated by the endosomal sorting complex required for transport (ESCRT) proteins, which are known to mediate a diverse array of membrane remodelling activities [3]. At its thickest point, the midbody diameter is 25 times greater than the estimated threshold for membrane remodelling by ESCRT proteins; Therefore, present models suggest that so-called secondary ingression is required to further thin the midbody bridge prior to membrane scission by the ESCRTs [4,5] (shown schematically in Figure 1).

Many studies have established that membrane trafficking plays a significant role in abscission as secondary ingression places heavy demands on membrane delivery to the abscission site [6]. McDonald and Martin-Serrano have presented a model that describes abscission in a series of stages [7]. First, Rab GTPases mediate the transport of post-Golgi and endosome-derived vesicles to the midbody, where they are targeted to specific sites on the plasma membrane by the Exocyst tethering complex [8,9]. Assemblies of septin filaments are proposed to outline compartments in the plasma membrane to restrict movement of the Exocyst, thereby promoting targeted vesicle delivery to the abscission site [10]. In the second stage, vesicle fusion with the plasma membrane is mediated by the interaction of the SNARE proteins endobrevin and syntaxin-2 [11]. In support of this model are data demonstrating fusion of these vesicles with the plasma membrane of the bridge occurring clearly before the final cleavage event [9]. For detail of these early stages of cytokinesis, the interested reader is referred to [6,12]. Finally, the arrival of CEP55 at the midbody recruits the ESCRT machinery for membrane scission [13] (Figure 1).

## 2. Endosomal Sorting Complex Required for Transport (ESCRT) Proteins

ESCRT proteins were identified in budding yeast as Class E *Vps* gene products and are known to have important roles in the membrane remodelling, which accompanies several processes, such as the membrane protein sorting which occurs during the down-regulation of ubiquitin-labelled receptors via the formation of multivesicular bodies (MVB), and in human immunodeficiency virus (HIV) budding from host cells [7]. The ESCRT machinery consists of four main protein complexes: ESCRT-0, -I, -II and -III, and an AAA-ATPase, VPS4, that disassembles the machinery [14]; For recent reviews of ESCRT biology, the interested reader is referred to [15,16]. Present models for MVB formation suggest that ESCRT-I and -II deform the membrane to form buds, ESCRT-III cleaves the buds, and VPS4 recycles ESCRT-III for additional rounds of budding [17]. Central to all of these activities is the ability of the ESCRT complex to drive membrane scission.

ESCRT-III has been identified as the primary driving force in membrane scission events [18]; precisely how this is achieved remains the subject of some debate, with a range of models posited. Using electron microscopy, the Hanson laboratory identified circular arrays of filaments on the plasma membranes of cells overexpressing ESCRT-III proteins [19]. Similarly, the Hurley laboratory showed, in an elegant series of reconstitution experiments on giant unilamellar vesicles, that ESCRT-III proteins were sufficient to drive intralumenal vesicle formation; Interestingly, they found that VPS4 was required to recycle the ESCRT-III components, but not the formation of the vesicles [18]. Such studies led to the idea of spirals of ESCRT-III filaments driving the scission event. In a subtle variation of this model, others have proposed that as ESCRT-III pinches membranes, they form a dome-shaped structure [20]. A further model was proposed by Saksena and colleagues who proposed that ESCRT-III binds directly to the ESCRT-II complex and oligomerises directly on the membrane into a filament; This led these workers to suggest that ESCRT-III forms a ring that is disassembled by the action of VPS4 at one end, with the net effect resembling a purse-string construction [21]. These different models all agree that ESCRT-III is intimately linked to scission, and that VPS4 is required both to redistribute ESCRT-III subunits back into the cytoplasm and has been shown to be necessary for all ESCRT-mediated processes [22,23].

Ground-breaking findings placed the ESCRT proteins at the centre of the abscission process [24,25]. Tumour susceptibility gene 101 (TSG101), an ESCRT-I component, and apoptosis-linked gene 2-interacting protein (ALIX) were shown to localise to the midbody during cytokinesis via interaction with CEP55 [13], and function with downstream ESCRT pathways members in membrane fission (Figure 1). The interaction between ALIX and ESCRT-III was demonstrated to be essential for cytokinesis, as disruption of this module, or RNAi-mediated depletion of either TSG101 or ALIX, inhibited abscission [24,25]. These findings were supported by inferences from the mechanistic similarities between established ESCRT-mediated membrane scission events and cytokinesis: MVB biogenesis and HIV virion egress both require the internal resolution of a cytoplasm-filled membrane tubule; Such an event is topologically opposed to that mediated by dynamin, but is similar to the final scission step of cytokinesis [15,19].

Regardless of mechanism, the overwhelming body of recent evidence has placed ESCRT and ESCRT-associated proteins, such as VPS4, at the heart of the abscission mechanism together with a growing list of ESCRT-associated proteins that also appear to play important roles in regulating aspects of ESCRT function, including cytokinesis (see for example [26,27,28]).

## 3. Function and Dynamics of ESCRTs at Cytokinesis

Understanding the function and dynamics of the ESCRT complex in cytokinesis has been driven largely by image analysis approaches. Early studies used electron tomography to identify ESCRT-III-dependent filaments at the constriction site, consistent with an ESCRT-III polymer (see below) [29]. However, the subsequent use of super-resolution imaging revealed ESCRT-III also accumulates asymmetrically on one side of the midbody in late cytokinesis, and that this accumulation is immediately followed by abscission [5] (Figure 1, B-onwards). This raises the interesting question of the dynamics of ESCRT complex behaviour in the midbody, and how it is regulated. Recent data have begun to shed some new insight into this process.

It is generally agreed upon that the ESCRT-I component TSG101 accumulates as two cortical rings adjacent to the Flemming body in early cytokinesis via interaction with CEP55 (Figure 1, panel A) [13]. The arrival of TSG101 at this structure, controlled in part by the action of Polo-like kinase on the centrosomal protein CEP55 [30], is in turn followed by the accumulation of ESCRT-III components to form a pair of additional ring-like structures on both sides of the Flemming body [5,29]. The distribution of the ESCRT-III accessory protein VPS4 exhibits a striking overlap with ESCRT-III, suggesting that ESCRT-III may undergo some kind of VPS4-mediated remodelling at or near the Flemming body (a point discussed further below). The next key stage of ESCRT function is the appearance of ESCRT-III proteins at a point some distance away from the Flemming body. This area appears to be coincident with a site of further ingression of the midbody bridge, the so-called secondary ingression site (see Figure 1B) [5]. The formation of the secondary ingression site is a key feature of the abscission mechanism. Two different models of secondary ingression have been posited. While neither is mutually exclusive, they offer a divergent view of the relative importance of different facets of the terminal abscission event.

### 3.1. Endosomes Drive Secondary Ingression

Many studies have shown that recycling endosomes accumulate in the intercellular bridge and are required for abscission. Detailed analysis of the arrival of the endosomes and ESCRT complex components has shown that the arrival of both ESCRT-III and recycling endosomes into the regions adjoining the Flemming body occurs early in telophase and precedes the formation of the secondary ingression zones (Figure 1C, insert) [6]. Work from the Prekeris laboratory has suggested that the recycling endosomes in the intercellular bridge drive the formation of the secondary ingression zones, and that only after the secondary ingression is established is the appearance of ESCRT-III (specifically CHMP4B) at the secondary ingression zone clearly manifest [31]. In their model, the high degree of curvature generated by the secondary ingression, perhaps coupled to lipid remodelling activities of enzymes known to associate with endosomes [32], acts as a focus for ESCRT-III polymerisation. Consistent with such a model, others have shown that ESCRT-II and CHMP6 act to localise ESCRT-III polymerisation on regions of high membrane curvature *in vitro*, and ESCRT-III function in MVB formation and viral egress is known to generate vesicles over two orders of magnitude smaller than the intercellular bridge [33].

### 3.2. ESCRTs Drive Secondary Ingression

High-resolution analysis of the midbody revealed electron-dense ripples on either side of the midbody [29]. This observation, together with super-resolution imaging that revealed CHMP4B apparently extending from the midbody rings towards the abscission sites, suggested that polymers of ESCRT-III may extend outwards from the Flemming body, and that this polymerisation may drive the secondary ingression. However, recent work from several groups suggests that continuous filaments of ESCRT-III components are not readily observed [31,34], begging the question of how ESCRT-III might reach the abscission site.

Elia *et al.* have used computational modeling to propose that abscission is driven by an ESCRT-III-dependent fission complex formed as a result of ESCRT-III polymerisation from the midbody rings assisted by the action of the ATPase VPS4, shown schematically in Figure 1C [34]. At the heart of this model is a biophysical/computational analysis of ESCRT dynamics. The model proposes that the initial ESCRT-III “ring” in the midbody (diameter ~1.25 µm) is inherently different from the favoured structure of ESCRT-III spirals (~50 nm). This mismatch is proposed to generate mechanical stress on the emerging ESCRT-III polymer, which facilitates a VPS4-mediated breakage of the polymer into two parts: one which remains associated with ESCRT-I at the midbody rings, and the other (which they propose is the fission complex) relaxes to its spontaneous diameter, generating a narrow cylinder with a dome-cap at the end (Figure 1C) [34]. Elia and colleagues theorise that the interaction of these ESCRT-III structures with the plasma membrane, mediated by a specific set of lipids in the intercellular bridge (see insert to Figure 1), drives strong deformation of the membrane. The latter structure may move to a pre-determined site (perhaps controlled by lipid microdomains) or alternatively the position of this structure may be determined by the elastic energy of the intercellular bridge dictating an “equilibrium position” where abscission takes place [34].

Several lines of evidence support this model. First, real-time imaging of the distribution of ESCRT-III proteins revealed that the peripheral ESCRT-III pool moved further from the central pool as abscission progressed, until a conserved distance of ~0.7 µm was reached. After this, no further “tightening” of the microtubule bundle altered this position, suggesting that an equilibrium position has been reached. This notion of an equilibrium position is also supported by a theoretical analysis, based upon known membrane bending energies and the Helfrich formula which support the notion of an equilibrium position between 0.6 and 0.8 µm, a figure remarkably close to that experimentally determined by analysis of cells in abscission [34]. A final and relevant twist to this model comes from the unexpected finding that the values of membrane affinity required at the dome neck to complete fission are more than an order of magnitude less than those measured *in vitro* (ε ~0.07 *versus* 3.45 mN/m, respectively). Perhaps the most persuasive element of this model is the observation that depletion of ATP (and by extension inhibition of VPS4 function) resulted in the appearance of an ESCRT-III polymer extending a significant distance from the midbody ring [34]. Thus, the role of VPS4 in ESCRT-III function in abscission is clearly important. This may explain why Prekeris and colleagues reported that CHMP4B oligomers, extending from the midbody to the abscission site were evident in only 27% of cells imaged [31].

### 3.3. Integrated Models

Recent work from our group has suggested that ESCRT and endosome function are closely linked, at least early in cytokinesis [35]. The arrival of CEP55 and ALIX in the midbody is dependent on the function of the endosomal SNARE syntaxin 16 (Sx16). We found that Sx16 is required both for the accumulation of recycling endosomes in the midbody and also for the midbody accumulation of CEP55 and ALIX, suggesting that Sx16-dependent trafficking is required for the correct placement of both endosomes and ESCRT machinery during early cytokinesis, and that these two distinct facets are tightly integrated. By extension, it would seem logical that similar intertwined mechanisms may be involved in the establishment of the abscission zones. Although the model of Elia *et al.* is compelling, some facets could also encompass an endosome-dependent component. For example, the equilibrium position could be defined by the presence of a lipid domain (or lipid remodelling) driven by endosomes in the bridge acting as platforms for such processes (insert to Figure 1C). Consistent with this are the observations that the lipid environment of the intercellular bridge is regulated and distinct from the rest of the plasma membrane, and that Rab35-dependent delivery of lipid remodelling enzymes are required for cytokinesis (insert to Figure 1C) [32,36]. This could also be germane to the need to sever microtubule bridges prior to fission. Recent work has shown that CHMP1B, a component of the ESCRT-III complex recruits the microtubule-severing enzyme spastin to the midbody via a non-canonical MIT-binding domain within spastin and the *C*-terminus of CHMP1B, providing further support to the idea of integration of multiple mechanistic events [37,38]. Work from the Prekeris group reveals that a key function of endosomes is to deliver p50RhoGAP into the abscission sites to regulate the cortical actin network [31]. One can imagine how these processes of actin regulation, endosome-dependent lipid remodelling and ESCRT-III movement are all inter-dependent.

## 4. ESCRTs in Other Organisms

The majority of the data published thus far examines the role of ESCRTs in cytokinesis in mammalian cells. An increasing body of evidence supports a role for ESCRTs in cell division in other organisms (see Table 1 for details of the nomenclature in these different systems). Here, we briefly review these studies.

**Table 1:** Details of ESCRT genes in different organisms.

ESCRT Class	Mammalian Cells	Fission Yeast	Budding Yeast	Worms	Arabidopsis	Archaea	Key Domains	Function
ESCRT-0	HRS	Sst4 (Vps27)	Vps27	Vps27			Ubiquitin binding domain, phosphatidylinositol 3-phosphate binding domain	Clustering of ubiquitylated cargo
	STAM1/2	Hse1	Hse1					
ESCRT-I	TSG101	Sst6 (Vps23)	Stp22	TSG-101	elc		Proline rich linker region that targets ESCRT-I to the midbody during cytokinesis	Membrane budding (with ESCRT-II) and cytokinesis
	Vps28	Vps28	Vps28					
	Vps37A, Vps37B, Vps37C	Vps37	Vps37					
	MVB12A, MVB12B		Mvb12	MVB-12				
ESCRT-II	EAP30	Dot2 (Vps22)	Snf8				Connecting MVBs to microtubules	Membrane budding (with ESCRT-I)
	EAP20	Vps25	Vps25					
	EAP45	Vps36	Vps36					
ESCRT-III	CHMP2A, CHMP2B	Vps2 (Did4)	Did4				MIM1/2 domain recruits Vps4 to initiate ESCRT-III disassembly. Winged helix domain	Membrane scission/cytokinesis
	CHMP3	Vps24	Vps24			Sacil 373		
	CHMP4A, CHMP4B, CHMP4C	Vps32 (Snf7)	Snf7	Vps32				
	CHMP6	Vps20	Vps20					
Vps4	Vps4A, Vps4B	Vps4	Vps4		Skd1	Sacil 373	MIT ESCRT-III binding domain, ATPase domain	ESCRT-III disassembly, cytokinesis MVB biogenesis
	CHMP5	SPCC162.06c	Vps60					
	Vta1	Vta1	Vta1					
ALIX	ALIX	Bro1	Bro1				Interacts with apoptosis factors and cytoskeleton. Recruit ESCRT-III to midbody	Targeting functions, cytokinesis/membrane abscission

### 4.1. Yeast

ESCRT proteins were first characterised in the budding yeast *Saccharomyces cerevisiae*, but their role in cytokinesis has only been relatively recently characterised [39]. Although ESCRT proteins have not been shown to localise to the bud neck, the site of cytokinesis in this organism, nor has a direct role for ESCRT proteins in abscission been demonstrated, evidence is emerging that they appear to have a role in cytokinesis. ESCRT-III *SNF7*^+^, when mutated, causes defects in cytokinesis, and exacerbated synthetic phenotypes are observed when *snf7* mutants are combined with mutants in *cla1* or *elm1* in which septin assembly is perturbed, an essential part of cytokinesis. Similarly, *snf7* mutants suppress temperature-sensitive mutant phenotypes of *CDC10*, which encodes septin [39]. Furthermore, the observation that chitin synthase II levels are elevated in *elm1 ESCRT* double mutant implies a role for ESCRT proteins in the recycling of proteins required for cytokinesis. Indeed, present studies have suggested that the main role of ESCRT proteins in budding yeast cell division is via effects on membrane trafficking. Regardless of mechanism, the potential control of ESCRT proteins by cell cycle regulators has been suggested by the observation of phosphorylation *in vitro* by polo kinase of Snf7 and Vps20—A point discussed further below [40].

ESCRTs have also been identified in the fission yeast *Schizosaccharomyces pombe* [41] and their role in cytokinesis established [38]. We observed that mutants in the various classes of ESCRT genes all cause cytokinetic defects, implying that they have a role in this process [40]. In contrast to the situation in budding yeast, however, GFP-tagged ESCRT proteins appear to localise close to the division septum, hinting at a direct role of ESCRTs in cell division (Kaupisch, Bhutta, Gould and McInerny—Unpublished data). Excitingly, a subset of ESCRTs interact genetically and physically with Plo1 and Aurora kinases and Cdc14p phosphatase, cell cycle regulators with established and important roles in cytokinesis. In some cases these interactions have been replicated in human cells with, for example, CHMP2 (Vps2p), CHMP3 (Vps24p), CHMP4 (Vps32p), and CHMP6 (Vps20p) co-immunoprecipitating with human PLK1 [40]. However, it is important to note that Bhutta *et al.* have also revealed an effect of Plo1 in membrane trafficking in *pombe*, and, thus, direct contributions of the ESCRT proteins to the abscission machinery are difficult to disentangle from effects mediated via membrane trafficking.

### 4.2. Worms

The role of ESCRT proteins in abscission in has been analysed in *Caenorhabditis elegans* with a role established for the ESCRT-I genes TSG-101 and MVB-12 [42]. It is proposed that abscission occurs in two stages, with the first involving cytoplasm isolation between two daughter cells, and the second the release of the midbody and midbody ring. Whereas inhibition of septins effects both stages, it appears that the ESCRT-I proteins are only required for the second stage. As *C. elegans* lacks CEP55 homologues it would seem that other molecules might recruit the ESCRT machinery to promote scission, potentially either centralspindlin or other midbody components. The identification of these factors may offer unique insight into mechanism and regulation.

### 4.3. Fungi

Recent work has identified a series of physical and genetic interactions between the key mitotic kinase never in mitosis a (NIMA), a member of the Nek family of protein kinases, and ESCRT proteins [43]. These interactions were shown to be required for normal polarised tip growth in *Aspergillus nidulans*, and give rise to the notion that mitotic kinase control of ESCRTs may be widespread across evolution, a point we shall return to below. The functional role of these events in the cell cycle remains to be described.

### 4.4. Plants

Analysis of the functional role(s) of ESCRT proteins in plants is restricted to only a few studies. Analysis of gene expression profile, proteomic and massively parallel signature sequencing projects has revealed a broad expression of all ESCRT components across tissues and stages of plant development [44,45]. Thus far, studies of ESCRT function in cytokinesis in plants have been limited to the *elc* mutant of *Arabidopsis thaliana* [46]. This mutant was originally identified via trichome morphogenesis mutant phenotype. However, further studies revealed that *elc* mutants are multinucleated in all endoreduplicating cell types studied, strongly suggesting a role for this gene product in cell division. Interestingly, *elc* encodes a TSG101 homologue and was shown to interact with *Arabidopsis* VPS37 and VPS28, other ESCRT-I components [46]. How *elc* functions in cell division in plants remains unclear. Spitzer and colleagues have suggested that this may arise via control of nuclear divisions through the cytoskeleton. The same group have defined an important role of the ESCRT-III proteins CHMP1A and B in plant embryo and seedling development [47]; Similar data were also reported for other components of the ESCRT-III pathway [48]. Whether this developmental defect arises through effects on endosomal sorting and trafficking (as has been suggested for *S. cerevisiae*—See above) or via mechanisms akin to those operating in mammalian cells remains to be defined. In a similar vein, studies of mutations in a novel VPS4 (*Skd1* in Arabidopsis) regulator (PROS) revealed profound effects on development and cell expansion [49]. Although in their infancy, studies of ESCRT mutations on plant cell division offer the potential to reveal new insight into the function of this class of proteins.

### 4.5. Archae

The modular nature of ESCRT components suggests divergent functions, both within particular cells and between organisms and even branches of evolution [50]. Studies in *Archaea* have revealed roles for ESCRT-III and VPS4 in cell division and potentially viral budding [50,51]. Both ESCRT-III and VPS4 were localised to the midcell during cell division, together with CdvA, the protein that recruits ESCRT-III to the membrane, where they form a cytokinetic ring [52]. This finding was particularly significant because *Archaea* lack endomembrane structures, thus, strengthening the view that ESCRT proteins have a well-conserved function in the scission phase of cytokinesis, rather than endosomal sorting for vesicular fusion. Recent work has demonstrated the formation of ESCRT filaments in the archaeon *Sulfolobus acidocaldarius* and point to an ancient mechanism by which ESCRT proteins form spirals for membrane scission and provide compelling support for the spiral/dome scission models described above [53].

## 5. ESCRT Regulation

### 5.1. Lipids may Control ESCRT Function

The timing studies performed by the Lippincott-Schwartz group aptly describe a mechanism for ESCRT-mediated abscission; However, several further questions are raised, perhaps most notably, the mechanism of CHMP4B nucleation into spirals. Elia and colleagues [34] suggest the involvement of lipid interactions, due to the proposed role of membrane trafficking in secondary ingression formation and the *in vitro* preference of ESCRT-III for acidic membranes [19,35]. They propose, therefore, that attractive forces mediated by the lipid bilayer composition may drive ESCRT-III to the constriction site [34]. It is worth noting that ESCRT components have been demonstrated to bind membranes in MVB formation and HIV budding via interactions with phosphatidylinositol 3-phosphate or cholesterol-rich lipid raft domains, respectively [4,23,54,55], and that such enriched domains have been reported to accumulate in the midbody [36,56].

Such lipid-mediated control nodes for ESCRT-III raise some interesting evolutionary questions, as *Archaea* cell membranes have quite distinct lipid composition and membrane structure. *Archaea* membranes are characterised by the presence of glycerol-1-phosphate linked by an ether to a range of phytanyl lipids (eukaryotes couple fatty acids to glycerol-3-phosphate by ester links). Recent studies in Archaea have shown that CdvA, the Archaeal protein that recruits ESCRT-III to membranes, is capable of polymerisation into helical filaments that wrap around liposomes, and can form nested tubes and cone-shaped structures, suggesting that these proteins are capable of forming a range of assemblies that may offer versatility and complexity to their function [53]. Most excitingly, the use of electron cryotomographic imaging in *S. acidocaldarius* revealed a protein “belt” at the leading edge of the construction furrows of dividing cells [53]. Although we await definitive proof that this belt is comprised of ESCRT proteins, the data are provocative and lend support to the models outlined above and hint that lipids may serve a crucial role in the assembly and function of the ESCRT machinery in all its functions. Although an interesting proposition, it seems unlikely that lipid composition in isolation could mediate ESCRT-III nucleation, though it may aid ESCRT-III translocation coincidentally with nucleation by another faction. Further insight into this possibility is provided below (see ANCHRs away).

### 5.2. Polo-Like Kinase and the Control of Early Cytokinesis

ESCRT components are phosphoproteins and phospho-regulation is emerging as an attractive direction of investigation [57]. Abscission is tightly controlled in space and time, and is also intricately coupled to previous steps in the cell cycle to ensure the fidelity of the cell division process. Polo-like kinase (PLK1) [58] and Aurora B kinase [59] have both emerged as exciting candidates to coordinate abscission events via direct effects on the abscission machinery [57]. PLK1 activity is high early in the cell cycle, but begins to decline in anaphase as PLK1 is degraded; This is a crucial early step in the commitment to cytokinesis and abscission, as the PLK1-dependent phosphorylation of CEP55 prevents its accumulation in the midbody [30]. As PLK1 activity falls, so CEP55 accumulates in the midzone and begins to orchestrate the assembly of the abscission machinery [13]. In their early work characterising ESCRT dynamics at the midbody, the Sundquist group demonstrated that CEP55 depletion results in failed accumulation of late-acting abscission factors at the midbody, including ESCRT proteins, Aurora B kinase and Plk1 [25]. Furthermore, inhibition of PLK1 activity was found to result in aberrant midbodies similarly lacking ESCRT proteins, VPS4, and other abscission components—A point we shall return to below [30,60].

### 5.3. Aurora Kinase and the Abscission Checkpoint

Aurora B activity is also known to fall during cytokinesis, and Aurora B at the midbody is inactive. The role of this kinase in regulating abscission progression has recently received considerable attention with the realisation that ESCRT proteins, specifically the ESCRT-III subunit CHMP4C, are direct targets of this kinase.

Premature inactivation of Aurora B promotes abscission, an observation which led to the suggestion that an Aurora B-mediated checkpoint exists to regulate entry into abscission—The NoCut checkpoint [61]. When chromatin is present within the intercellular bridge, abscission must be delayed to allow this chromatin to be removed; This serves to prevent aneuploidy in cells with chromosome separation defects and also facilitates DNA damage repair. This abscission delay is achieved by a sustained activation of Aurora B kinase; A key target of this kinase is CHMP4C [62]. Depletion of CHMP4C using siRNA decreased abscission time in HeLa cells by around 30 min. Over-expression of CHMP4C restored abscission kinetics, but over-expression of a phospho-resistant mutant of CHMP4C did not, leading Carlton and colleagues to propose that CHMP4C inhibits abscission upon phosphorylation by Aurora B [62]. Phosphorylation of CHMP4C was shown to drive its localisation to the Flemming body where it activates the NoCut pathway. In mammals, this phosphorylation occurred at residue S210 in a consensus site present only in mammals. Strikingly similar conclusions were reached by D’Avino and co-workers who identified a direct interaction between Borealin (a chromosomal passenger complex component) with Snf7 components of ESCRT-III in both humans and flies [63]. They, also, identified Aurora B-dependent phosphorylation of CHMP4C in the carboxy terminus, and speculate that these interactions and phosphorylations may serve to control the polymerisation and function of ESCRT-III during abscission. In their model, Aurora B phosphorylation of CHMP4C “holds” this protein in a conformation capable of binding borealin and unable to associate with membranes or other ESCRT-III components. Declining activity of Aurora B removes the interaction with borealin and allows ESCRT-III to associate with membranes and self-associate [63]. How these events, which presently require direct experimental proof, couple to the dynamics of ESCRT-III components described above remains a major target for research within the field.

### 5.4. ANCHRs Away

Further molecular detail of potential ESCRT control mechanisms has been revealed by studies from the Stenmark lab who identified a phophatidylinositol 3-phosphate binding protein called ANCHR (Abscission/NoCut Checkpoint Regulator); Over-expression of ANCHR prevents abscission, and its depletion decreased abscission time, suggesting ANCHR functions as a negative regulator of abscission [64]. ANCHR was shown to function as part of the Aurora B-dependent abscission checkpoint. Insight into the mechanism involved was provided by the identification of an association between ANCHR and the ESCRT regulatory ATPase VPS4. These proteins colocalise at the centrosome in interphase and early in mitosis, but in cytokinesis ANCHR appears to localise at the Flemming body, whereas VPS4 localises to the midzone spindle, an area adjacent to the midbody. Careful image analysis however revealed that VPS4 occasionally localised with ANCHR at the Flemming body, and that this localisation was dramatically increased in cells with lagging chromosomes in the intercellular bridge (*i.e.*, cells with the Aurora B mediated abscission checkpoint activated). Hence, they postulated that VPS4 interacts with ANCHR at the midbody ring in a manner regulated by Aurora B. As Aurora B activity falls, this interaction is released and VPS4 then acts at the abscission site to mediate ESCRT-dependent abscission [64]. In a pleasing synthesis of models, the Stenmark group then showed that VPS4, ANCHR, and CHMP4C are capable of forming a ternary complex, but inhibition of Aurora B activity using a small molecule inhibitor greatly reduced this interaction. Hence, these authors proposed that Aurora B phosphorylates CHMP4C and thus facilitates interaction with VPS4 and ANCHR to “lock” this abscission complex in an inactive form on the midbody; As Aurora B levels fall, this complex is disrupted, ANCHR releases the ESCRT-III/VPS4 complex and abscission can begin at the secondary ingression site (see above). Such data offer a unique insight into the complex network of regulation involved in the NoCut pathway of abscission control [64]. When the NoCut pathway is activated, the high levels of Aurora B activity maintain this complex in an inactive state at the midbody until such time as cell division can be successfully completed.

### 5.5. Is There More

The data described above offer insight into the molecular basis of events late in abscission, and how abscission timing may be controlled; However, there may be further levels of complexity to this model. In budding yeast, Aurora B was shown to localise to the anaphase spindle due to the phosphatase activity of Cdc14 [65]. The less well-characterised human CDC14A is required for Rab5-mediated endocytic trafficking [66], and it is possible that such an association may provide a role for CDC14A in controlling membrane trafficking to the midbody during cytokinesis. Similarly, work from our lab has identified an important role for membrane trafficking in the placement of early cytokinesis molecules, including CEP55, ALIX and molecules involved in endosomal trafficking [35]. Whether these events are similarly controlled, and if so by what route remains unclear. Unravelling these control nodes offers exciting potential for the field.

### 5.6. Plk and Abscission—Hints from Schizosaccharomyces pombe

Plk1 reappears on midbodies very late in cytokinesis, implying an abscission role for this kinase [60]. We identified genetic interactions between ESCRT proteins and polo and aurora kinases and Cdc14 phosphatase that manifest as impaired growth and exacerbated defects in septation, suggesting that the encoded proteins function together to control these processes [40]. These conclusions were supported by two-hybrid *in vivo* interactions between Plo1p and Sst4p, Vps28p, Vps25p, Vps20p and Vps32p and co-immunoprecipitation of human homologues of Vps20p, Vps32p, Vps24p and Vps2p by human Plk1 [40]. Interestingly, we were able to demonstrate direct *in vitro* phosphorylation of budding yeast Vps20p and Vps32p by polo kinase. Two-hybrid analyses also identified interactions between Ark1p (Aurora kinase) and Vps20p and Vps32p, and Clp1p (Cdc14 phosphatase) and Vps28p [40]. These experiments indicate a network of interactions between ESCRT proteins, *plo1*, *ark1* and *clp1* that coordinate membrane trafficking and cell separation in fission yeast. How these interactions work, and whether they are replicated in human cells, remains an open question.

## 6. Concluding Remarks

The role of ESCRTs in abscission is firmly established. How these interesting proteins functionally cooperate in space and time in response to defined signals remains only partly understood. One might bind together several of the concepts discussed here by proposing that alterations in phosphorylation state elicit ESCRT conformational changes that regulate interactions with other ESCRT subunits, ESCRT-interacting regulatory proteins (such as VPS4, Ist1p or others) or lipids. Nevertheless, it is clear that phospho-regulation, and the protein kinases and phosphatases that regulate these events, will form a significant component of the future research into ESCRT proteins.
